# MicroRNA-98 negatively regulates myocardial infarction-induced apoptosis by down-regulating Fas and caspase-3

**DOI:** 10.1038/s41598-017-07578-x

**Published:** 2017-08-07

**Authors:** Chuan Sun, Huibin Liu, Jing Guo, Yang Yu, Di Yang, Fang He, Zhimin Du

**Affiliations:** 1 Institute of Clinical Pharmacy, the Second Affiliated Hospital of Harbin Medical University (The University Key Laboratory of Drug Research, Heilongjiang Province), Harbin, 150086 China; 2 Department of Clinical Pharmarcology, College of Pharmacy, Harbin Medical University, Harbin, 150086 China

## Abstract

Acute myocardial infarction (MI) is the leading cause of sudden death worldwide. MicroRNAs (miRs) is a novel class of regulators of cardiovascular diseases such as MI. This study aimed to explore the role of miR-98 in MI and its underlying mechanisms. We found that miR-98 was downregulated both in infarcted and ischemic myocardium of MI mice as well as H_2_O_2_-treated neonatal rat ventricular myocytes (NRVCs). miR-98 overexpression remarkably increased cell viability and inhibited apoptosis of H_2_O_2_-treated NRVCs. Meanwhile, overexpression of miR-98 reversed H_2_O_2_-induced Bcl-2 downregulation and Bax elevation and significantly reduced JC-1 monomeric cells. Meanwhile, miR-98 overexpression attenuated the upregulation of Fas and caspase-3 in H_2_O_2_-treated cardiomyocytes at the mRNA and protein levels. Dual-luciferase reporter assay showed that miR-98 directly targeted to Fas 3′-UTR. Furthermore, MI mice injected with miR-98-agomir had a significant reduction of apoptotic cells, the serum LDH levels, myocardial caspase-3 activity, Fas and caspase-3 expression in heart tissues. Administration of miR-98-agomir also showed decreased infarct size and improved cardiac function. Collectively, miR-98 is downregulated in the MI heart and NRVCs in response to H_2_O_2_ stress, and miR-98 overexpression protects cardiomyocytes against apoptosis. Anti-apoptotic effects of miR-98 are associated with regulation of Fas/Caspase-3 apoptotic signal pathway.

## Introduction

Acute myocardial infarction (AMI), resulting from coronary artery occlusion, is the most common causes of cardiovascular morbidity and mortality worldwide^1^. Apoptosis, which is triggered by an imbalance between pro- and anti-apoptotic factors, is frequently detected in ischemic heart tissue^2^. Cardiomyocyte apoptotic death in the border area close to myocardial infarcted area leads to cardiomyocyte loss, aggravates cardiac dysfunction and even causes heart failure and mortality^3, 4^. Therefore, targeting inhibition of cardiomyocytes apoptosis during early stage of MI is critical for reducing infarct size and promoting cardiac repair, which is a key approach for treating ischemic heart disease. However, the molecular components regulating AMI-induced apoptosis in cardiomyocytes remain poorly understood.

MicroRNAs (miRNAs) are a group of small, endogenous and non-coding RNA molecules, with about 22 nucleotides in length, which has been shown to post-transcriptionally regulate the expression of target genes, leading to the destruction and degeneration of mRNAs^5^. Clinical trials and animal experiments indicate that miRNAs are potential biomarkers and therapeutic targets for cardiac ischemia^6, 7^. Moreover, several miRNAs are implicated in playing regulatory roles in cardiomyocytes apoptosis. Recent studies elucidate that miR-21, -24, -133, -210, -494 and -499 prevent myocytes against ischemia/reperfusion-induced apoptosis, while miR-1, -29, -195, -199a, -497 and -320 promote apoptosis^8–10^. Our previous study has also shown that combination of miR-21 and miR-146a has a greater protective effect against cardiac ischemia/hypoxia-induced apoptosis^11^.

MiR-98 is one of the members of the let-7 miRNA family, which is first discovered to control the developmental timing of cell differentiation and proliferation in C. elegans^12, 13^. Recently, altered miR-98 expression has been found in several carcinomas^14^. Therefore, let-7/miR-98 miRNAs are considered as an oncomir family crucial in regulating cell cycle and apoptosis^15^. Moreover, miR-98 has been reported to be a sensitive marker of renal ischemic injury^16^ and protect endothelial cells against hypoxia/reoxygenation induced-apoptosis by targeting caspase-3^17^. In addition, miR-98 was verified to target Fas directly and regulated Fas-mediated apoptosis in HeLa cells^18^. However, the functional roles of miR-98 in cardiomyocytes apoptosis during early stage of MI have not previously been investigated.

In the current study, we employed a mouse MI model and H_2_O_2_-induced cardiomyocyte injury model to investigate whether miR-98 had a protective effect against MI-induced cardiomyocytes apoptosis and myocardial dysfunction. Our findings suggest that miR-98 may provide a potential novel therapeutic approach for the treatment of ischemic heart disease.

## Results

### MiR-98 is downregulated in response to myocardial ischemic injury

**Figure 1:**
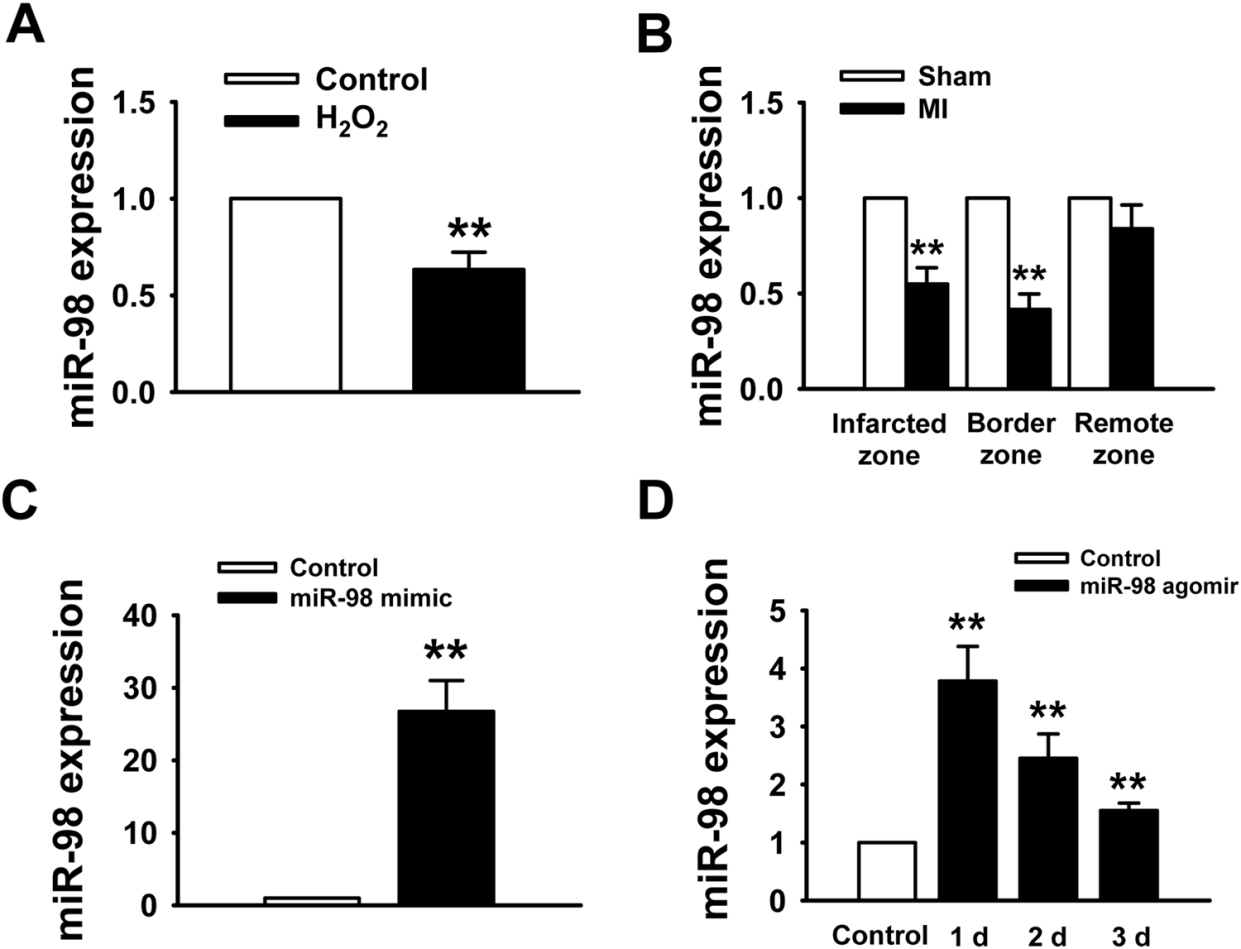
MiR-98 is downregulated in MI mice model and H_2_O_2_-treated NRVCs. (**A**) Real-time PCR data showed the downregulation of miR-98 expression in NRVCs treated with 100 μM H_2_O_2_ for 4 h. n = 6. (**B**) miR-98 is decreased in the infarcted and border zones of MI mice compared with sham mice. n = 3. (**C**) miR-98 expression level in NRVCs after miR-98 mimic transfection. n = 6. (**D**) miR-98 expression level in rat ventricles after administration with miR-98 agomir. n = 3. **P < 0.01 vs. Control or Sham.

Firstly, the expression of miR-98 was detected in H_2_O_2_-treated cardiomyocytes and postinfarct cardiac tissues. As our previous study^10^ reported that H_2_O_2_ reduced cardiomyocyte viability in a concentration and time-dependent manner, we chose 100 μM H_2_O_2_ treated NRVCs for 4 h as a cardiomyocyte injury model in this study. As shown in Fig. 1A, compared with control group, miR-98 expression was significantly decreased by exposure to 100 μM H_2_O_2_ in NRVCs. Meanwhile, we also examined the expression of miR-98 in heart tissues after MI for 3 days. To determine the change of miR-98 in the different areas of infarcted hearts, miRNAs were isolated from infarcted zone, border zone and remote zone. Real-time PCR analysis revealed that the expression of miR-98 in the infarcted and border zones of rat hearts at 3 days after MI was much lower than that in sham-operated animals (Fig. 1B).

**Figure 2:**
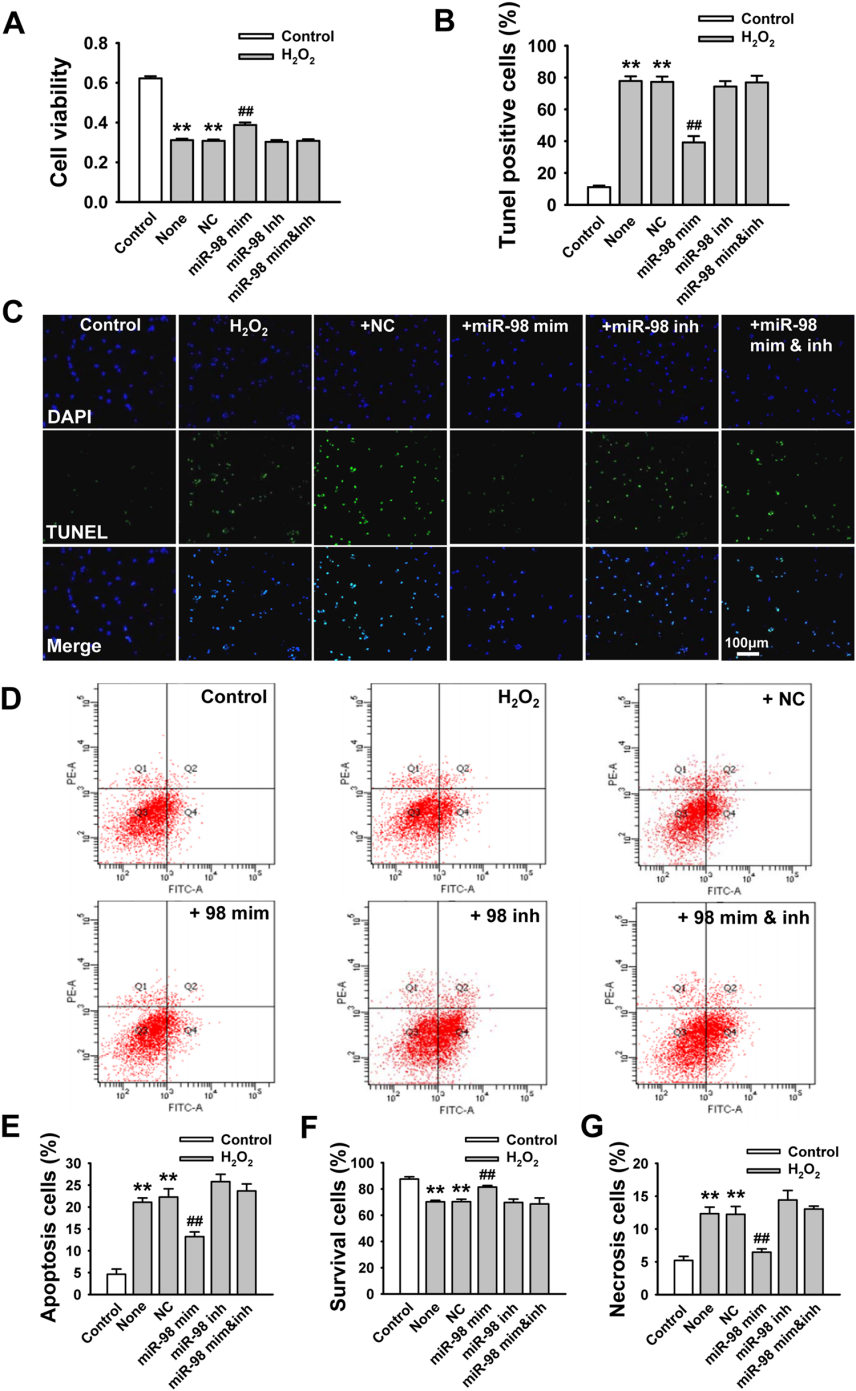
Overexpression of miR-98 prevents cardiomyocyte apoptosis in response to H_2_O_2_. NRVCs were transfected with NC (negative control), miR-98 mimic (miR-98 mim), miR-98 inhibitor (miR-98 inh), and miR-98 mimic + inhibitor (miR-98 mim&inh) and then treated with H_2_O_2_ (100 μM) for 4 h. (**A**) MTT assay suggested that miR-98 mimic restored cell viability of NRVCs treated with 100 μM H_2_O_2_ for 4 h. n = 8. (**B**) Statistical results of TUNEL-positive cells per field indicated that miR-98 mimic suppressed H_2_O_2_ treatment-induced cell apoptosis. n = 6. (**C**) Representative images of TUNEL staining of NRVCs for DNA defragmentation showing the apoptotic cells (nucleus stained in blue with DAPI and apoptotic cells stained in green). (**D**) The representative images of flow cytometry using Annexin V-FITC and PI staining. (**E**)-(**G**) Statistical analysis of apoptotic, survival and necrosis ratio of the flow cytometry data. n = 4; **P < 0.01 versus control; ^*##*^ *P* < 0.01 versus H_2_O_2_ + NC cells.

Then, we overexpressed miR-98 in NRVCs by miR-98 mimic transfection and in mice hearts by miR-98 agomir injection. We validated the miR-98 level using Real-time PCR, and found that miR-98 level was significantly higher in miR-98 mimic transfection group than that in non-transfection group (Fig. 1C). As shown in Fig. 1D, 1, 2 and 3 days after injection of miR-98 agomir using our delivery method, miR-98 expression was markedly increased in the heart tissue.

**Figure 3:**
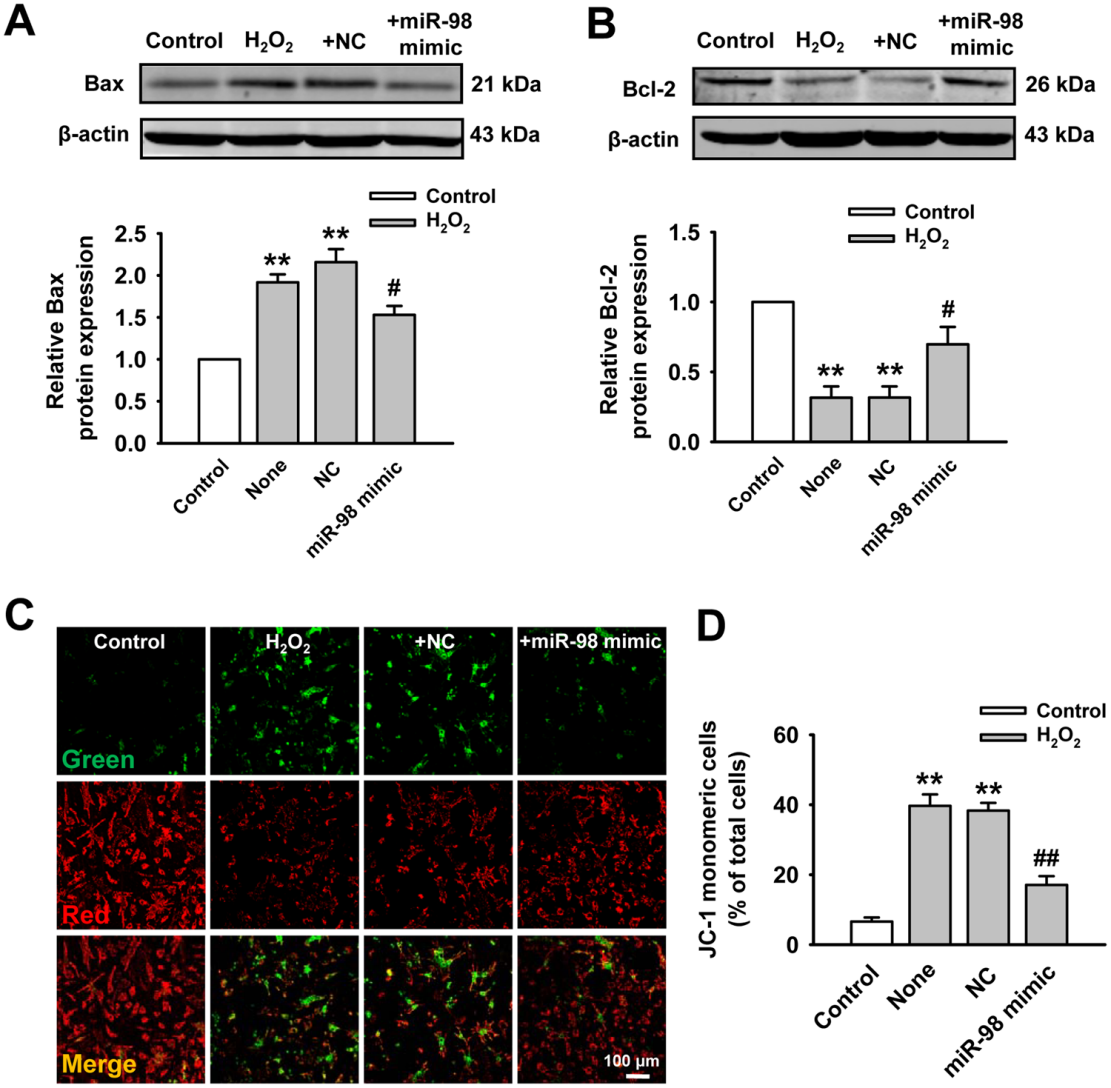
Effect of miR-98 on Bax and Bcl-2 expression and mitochondrial membrane potential (Δψm). (**A**) miR-98 overexpression reduced H_2_O_2_-induced elevation of Bax expression. Cropped blots are shown. Full-length blots are presented in Supplementary Fig. S1. n = 6. (**B**) Bcl-2 expression was suppressed by H_2_O_2_ but upregulated by miR-98. Cropped blots are shown. Full-length blots are presented in Supplementary Fig. S2. n = 6. (**C**) Upper panels show representative fluorescent images of JC-1 monomeric mitochondria showing green fluorescence and JC-1 aggregated mitochondria from Control, H_2_O_2_, H_2_O_2_ + NC and H_2_O_2_ + miR-98 mimic groups. (**D**) Overexpression of miR-98 reduced H_2_O_2_-induced JC-1 monomeric mitochondria. n = 5. **P < 0.01 versus control; ^*#*^ *P* < 0.05, ^*##*^ *P* < 0.01 versus H_2_O_2_ + NC cells.

### MiR-98 overexpression prevented H_2_O_2_-induced cardiomyocyte apoptosis

Based on the above results, we subsequently aimed to evaluate the effects of miR-98 overexpression on cell apoptosis. Cells subjected to 100 μM H_2_O_2_ for 4 h showed markedly decreased viability by MTT assay (Fig. 2A). In contrast, compared with miRNA negative control (NC) transfection group, miR-98 overexpression by transfecting with miR-98 mimic significantly increased the cell viability of NRVCs treated with H_2_O_2_ (Fig. 2A). Furthermore, it was observed that the number of TUNEL-positive cells was significantly increased in H_2_O_2_ group, which was diminished by miR-98 mimic but not by NC or miR-98 inhibitor (Fig. 2B and C). In addition, flow cytometry was utilized to validate the protective role of miR-98 in H_2_O_2_-induced cardiomyocyte apoptosis. We found that the apoptosis percentage was increased by H_2_O_2_ treatment, which was significantly diminished by miR-98 mimic (Figure 2 D and E).

Furthermore, the effect of miR-98 mimic on H_2_O_2_-induced cell apoptosis was abolished by co-transfection with miR-98 inhibitor (Fig. 2D and E). Moreover, overexpression of miR-98 also increased cell viability and prevented cell necrosis in H_2_O_2_-treated NRVCs (Fig. 2F and G). Taken together, these results proved that miR-98 overexpression prevented H_2_O_2_-induced cardiomyocyte apoptosis and promoted cell survival.

### MiR-98 overexpression regulates apoptosis-related proteins and mitochondrial membrane potential

Since miR-98 promoted cell survival and prevented cardiomyocyte apoptosis, we further investigated its role in regulating the expression of apoptosis-related proteins and mitochondrial membrane potential **(**Δψm**)**. Bax is pro-apoptotic protein which is absent/or very less in normal rat myocardium. As shown in Fig. 3A, exposure to H_2_O_2_ increased the Bax expression. However, miR-98 overexpression induced a sharp decrease of Bax expression in NRVCs compared with NC group (Fig. 3A). Antiapoptotic protein Bcl-2 was expressed in healthy control NRVCs. But exposure of NRVCs to H_2_O_2_ showed lower expression level of Bcl-2 than those in control NRVCs. Also, Bcl-2 expression was elevated in NRVCs after miR-98 overexpression (Fig. 3B). The corresponding densitometry analysis exhibited similar as the above descriptions (Fig. 3A and B).

Mitochondrial damage was determined using a mitochondrial membrane potential kit. The increase in the number of JC-1 monomeric cells (green) reflected the loss of Δψm. Compared with control cells, the number of JC-1 monomeric cells was remarkably increased in H_2_O_2_-stimulated cells. However, H_2_O_2_-induced increase in monomeric form cells was reduced by miR-98 overexpression (Fig. 3C and D).

### MiR-98 overexpression suppresses H_2_O_2_-induced upregulation of Fas and caspase-3 in cardiomyocytes

**Figure 4:**
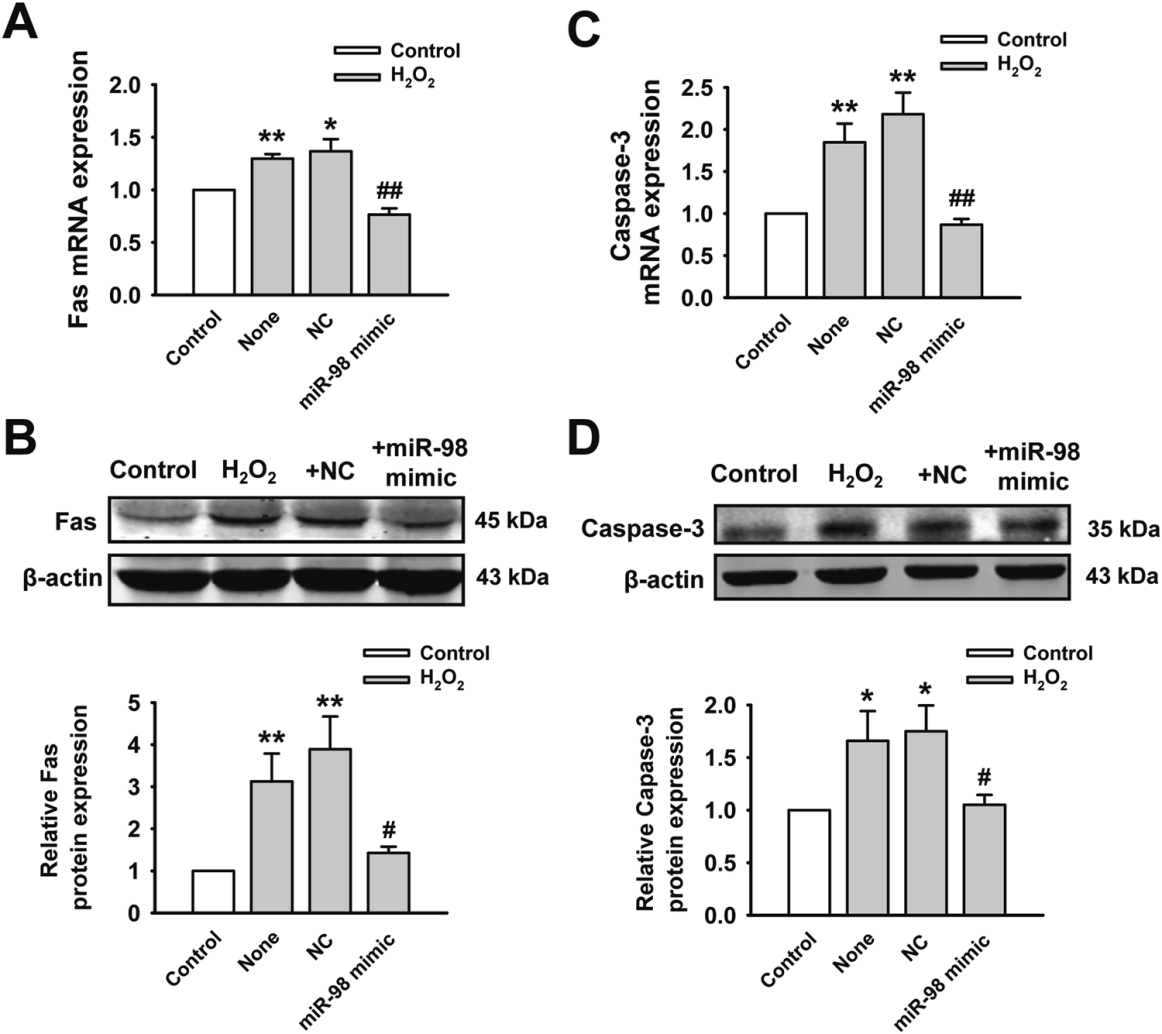
MiR-98 overexpression inhibits the expression of Fas and caspase-3. (**A**) and (**B**) MiR-98 overexpression significantly prevented upregulation of Fas mRNA and protein level in H_2_O_2_-treated NRVCs. Cropped blots are shown. Full-length blots are presented in Supplementary Fig. S3. n = 5. (**C**) and (**D**) The mRNA and protein expression of caspase-3 were also remarkably elevated by H_2_O_2_ but reduced by miR-98 overexpression. Cropped blots are shown. Full-length blots are presented in Supplementary Fig. S4. n = 6. *P < 0.05, **P < 0.01 versus control; ^*#*^ *P* < 0.05, ^*##*^ *P* < 0.01 versus H_2_O_2_ + NC cells.

We next aimed to explore the underlying mechanism that miR-98 inhibited H_2_O_2_-induced apoptosis. As Fas and caspase-3 were proved to be the target genes of miR-98^17, 18^, we further verified the expression of Fas and caspase-3 in the presence of miR-98 overexpression. As shown in Fig. 4A, compared with control group, Fas mRNA expression was significantly upregulated in the H_2_O_2_-treated NRVCs, which could be reversed by miR-98 overexpression. Furthermore, it is worth noting that the protein expression of Fas was markedly higher under H_2_O_2_ conditions than those in H_2_O_2_-free group. MiR-98 overexpression led to the decreased Fas protein expression in posttranscriptional level (Fig. 4B), further indicating that Fas was the target gene of miR-98. Meanwhile, miR-98 overexpression also reduced the upregulation of caspase-3 mRNA induced by H_2_O_2_ (Fig. 4C). The protein level of caspase-3 after miR-98 mimic transfection showed the similar trend with the mRNA level (Fig. 4D). Consequently, miR-98 could reverse the H_2_O_2_ induced elevation of Fas and caspase-3, and thus provide protections against ischemia-induced cardiomyocyte apoptosis.

### MiR-98 directly targets at the 3′-UTR of Fas

**Figure 5:**
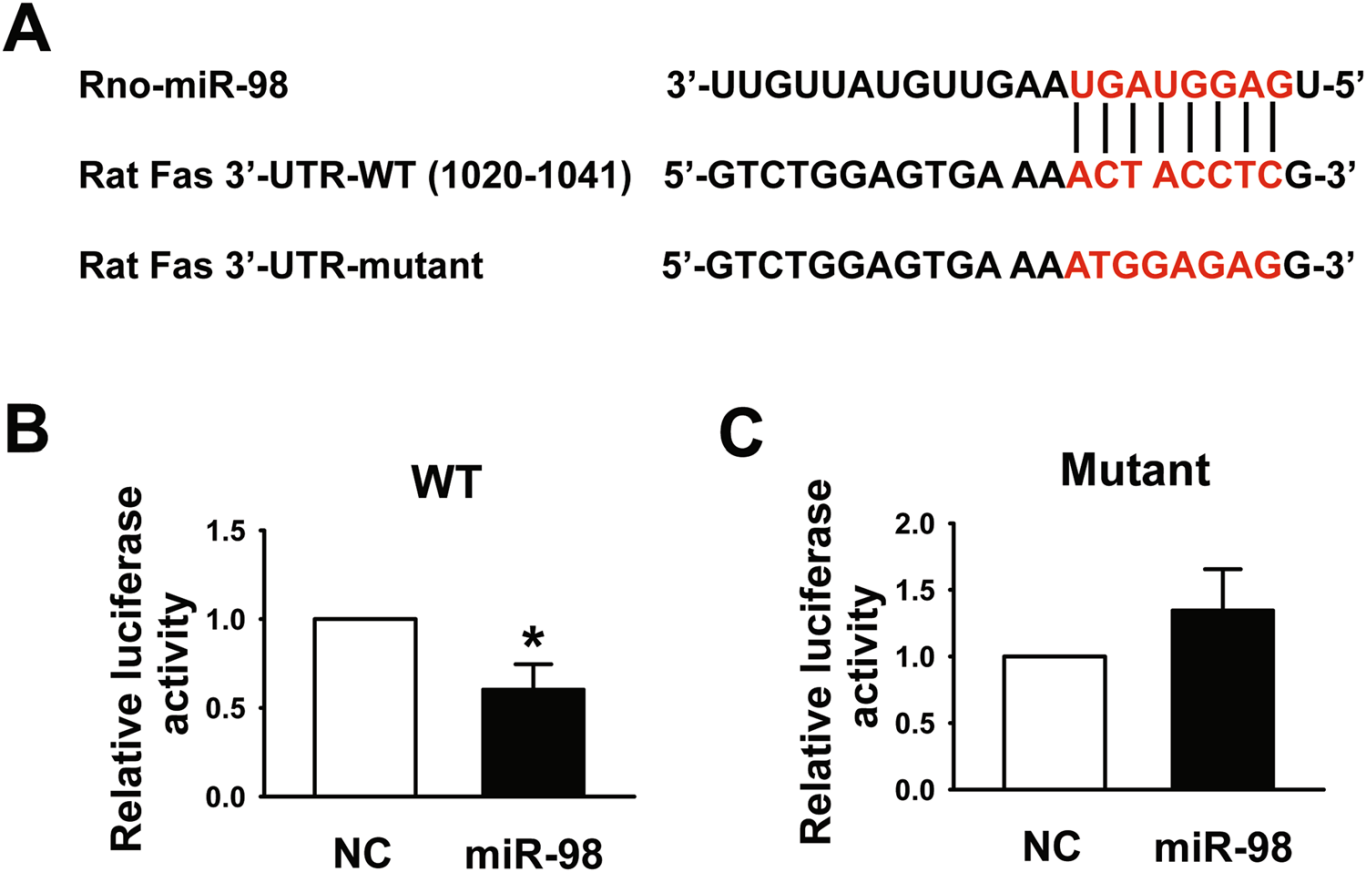
Fas was the target gene of miR-98. (**A**) The bioinformatic analysis showed that miR-98 had a binding site in the 3′-UTR of Fas mRNA and the mutation of the putative binding site was designed. (**B**) and (**C**) Dual Luciferase reporter assay was performed by co-transfection of luciferase reporter containing WT or mutant 3′-UTR of rat Fas with miR-98 mimic into HEK293T cells. MiR-98 overexpression markedly decreased the relative luciferase activity in the WT 3′-UTR but not mutant 3′-UTR of Fas mRNA. n = 6. *P < 0.05 versus NC miRNA.

Fas and caspase-3 were identified to be the target genes of miR-98 in humans^17, 18^ and the predicted site in caspase-3 3′-UTR showed a good conservative character among different species^17^. Due to the non-homology of Fas in different species, we used computational methods to search for the potential targets of miR-98 in rats and constructed luciferase reporter plasmids containing the 3′-UTR of Fas. The binding sites of miR-98 in the 3′-UTR of wild-type Fas mRNA were displayed, but mutant mRNA had few binding sites (Fig. 5A). We then transfected HEK293T cells with the luciferase vector containing a wild-type or mutant miR-98 response element. We cotransfected these cells with NC or miR-98 mimic and measured luciferase activity. miR-98 overexpression significantly inhibited luciferase activity in the wild-type group, demonstrating that miR-98 could target at 3′-UTR of Fas (Fig. 5B). However, miR-98 failed to affect the luciferase activity elicited by the construct carrying the Fas 3′-UTR with the mutant miR-98-binding site (Fig. 5C). Therefore, Fas was proved to be the target gene of miR-98.

### Effect of miR-98 overexpression on ischemia-induced cardiomyocyte apoptosis

**Figure 6:**
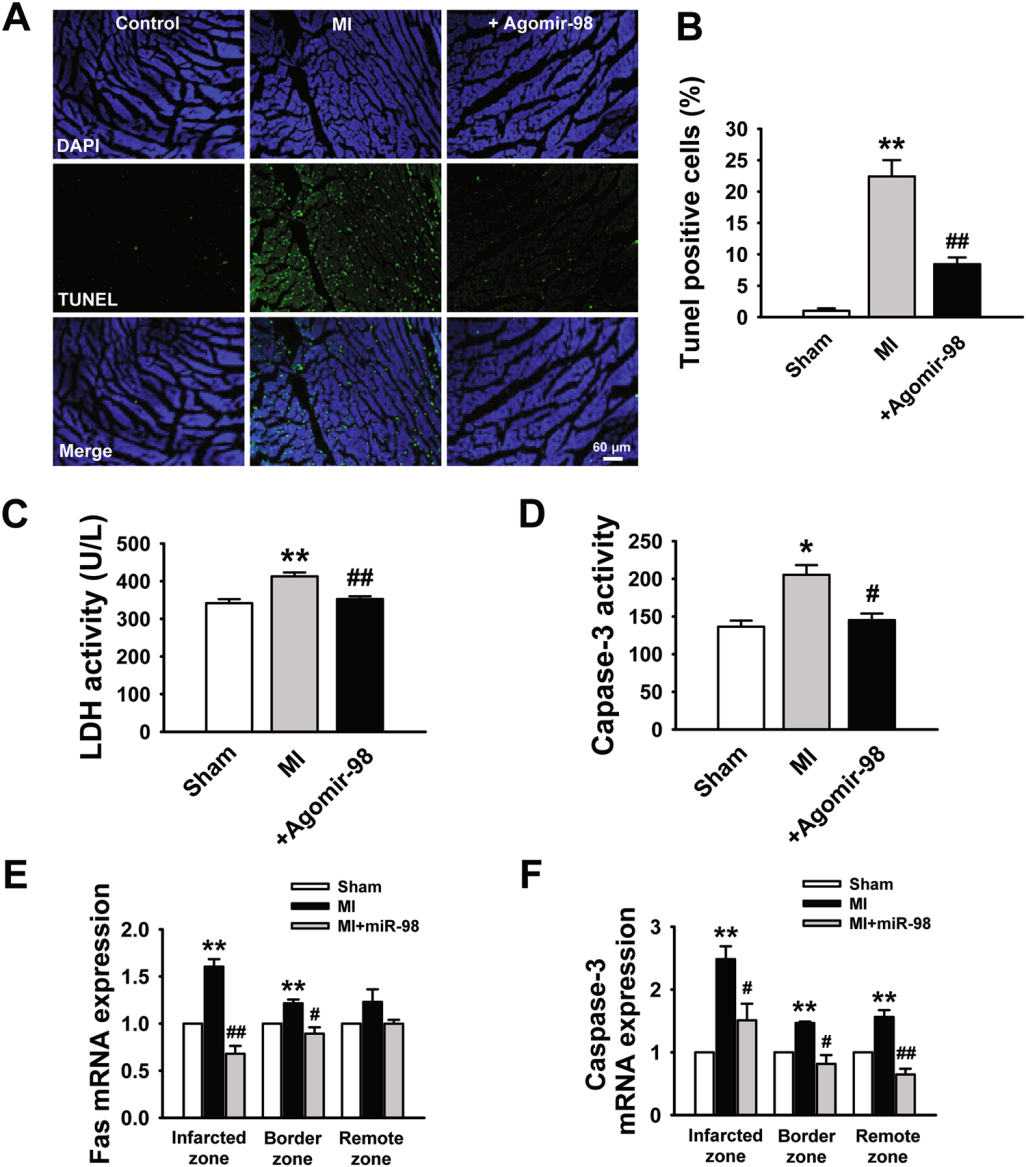
miR-98 protected cardiomyocytes against ischemia-induced apoptosis in a mouse MI model. (**A**) Effects of miR-98 agomir on cardiac apoptosis were evaluated by TUNEL staining. (**B**) The percentage of TUNEL-positive cell in different groups. n = 6. (**C**) Serum lactate dehydrogenase (LDH) activity is increased in MI mice and restored by miR-98 agomir administration. n = 6. (**D**) Caspase-3 activity is promoted in MI mice and reversed by miR-98 agomir. n = 5. (**E**) MiR-98 significantly prevented upregulation of Fas mRNA level in the infarcted and border zones of MI mice. n = 5. (**F**) MiR-98 suppressed the elevation of caspase-3 mRNA level in the infarcted, border and remote zones of MI mice. n = 5. *P < 0.05, **P < 0.01 versus sham group; ^*#*^ *P* < 0.05, ^*##*^ *P* < 0.01 versus MI group.

We then tried to clarify whether antiapoptotic effects of miR-98 on cultured cells under H_2_O_2_ conditions also exist under in *vivo* conditions in MI. Figure 6A and B showed that cardiomyocyte apoptosis significantly increased after MI, and treatment with miR-98 agomir, drastically decreased this ischemic apoptosis compared with that in the sham treated mice. Moreover, the activity of serum lactate dehydrogenase (LDH) (a marker for cardiac injury) increased obviously after MI, which was significantly attenuated by miR-98 agomir as well (Fig. 6C). Next, we measured the changes of expression and activity of caspase-3. As illustrated in Fig. 6D, MI increased the level of caspase-3 activity as compared to control group. As expected, this elevation of caspase-3 activity was blocked by miR-98 agomir administration. We also examined the expression of Fas and caspase-3 in different zones after MI for 3 days. Real-time PCR analysis revealed that Fas mRNA levels were markedly increased in infarcted and border zones and the existence of miR-98 agomir led to the decreased expression of Fas in transcriptional level (Fig. 6E). Meanwhile, caspase-3 mRNA levels were significantly increased in the whole heart, which could be prevented by miR-98 agomir (Fig. 6F).

### Overexpression of miR-98 decreases infarct size and improves cardiac function of infarcted heart in mice

**Figure 7:**
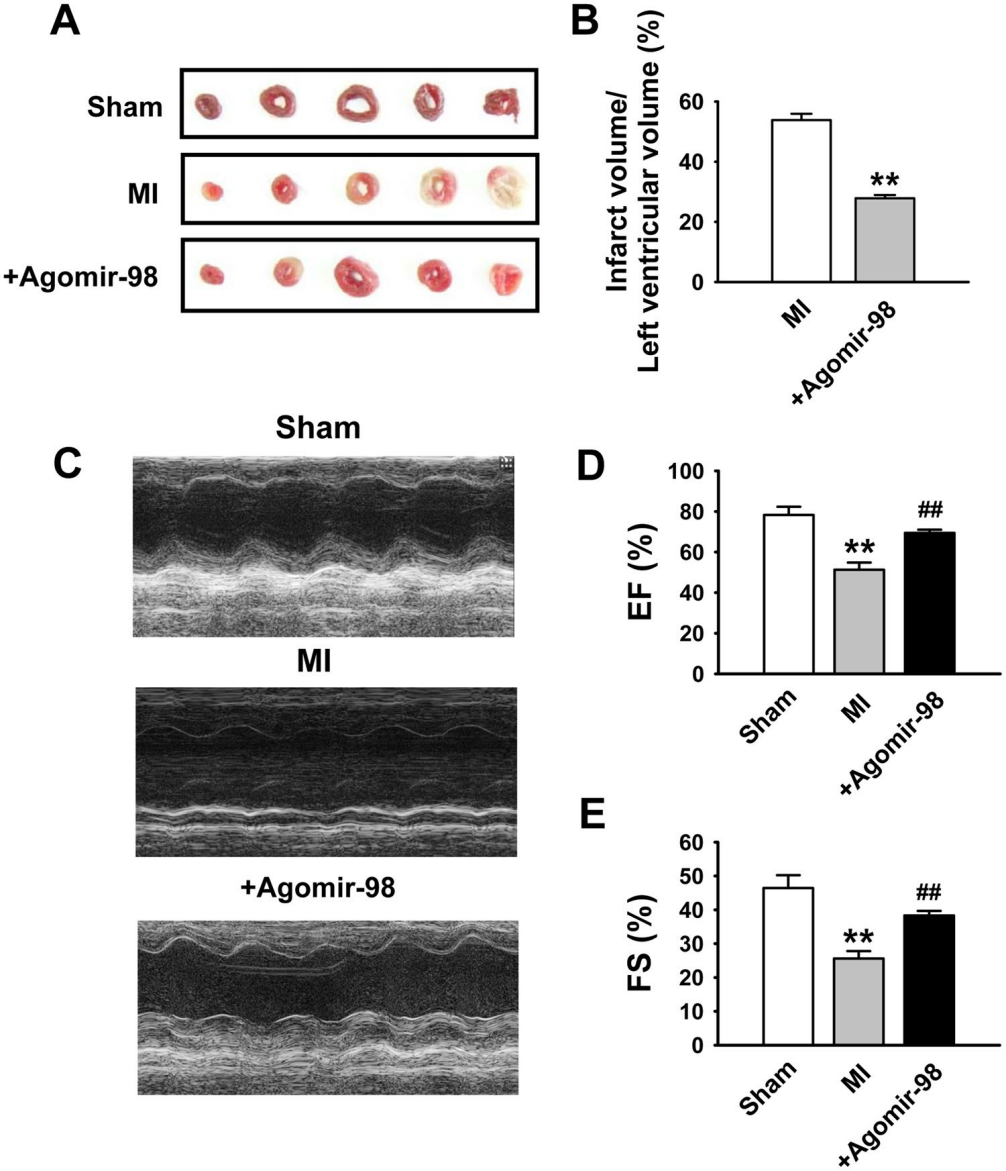
Reduction of infarct size and improvement of cardiac function by miR-98 in MI mice. (**A**) Representative images showing infarct areas in cross section slices. (**B**) Statistical analysis of IA/LV ratio. IA: infarct area, LV: left ventricles. n = 3. ﻿**P < 0.01 versus MI group. (**C**) Representative photographs of heart function. (**D**) Ejection fractions (**EF**) and (**E**) Fractional shortening (FS). n = 6. **P < 0.01 versus sham group; ^*##*^ *P* < 0.01 versus MI group.

We then investigated whether the beneficial effects of miR-98 exist in *in vivo* conditions. Before coronary artery ligation, miR-98 agomir was administered, which caused a continuous elevation of miR-98 (Fig. 1D). We detected the functional role of miR-98 agomir in infarcted heart and found that miR-98 agomir significantly reduced the infarct size in MI (Fig. 7A and B). Additionally, echocardiography examination showed that ejection fraction (EF) and fractional shortening (FS) were significantly decreased in MI hearts, indicating impaired cardiac functions (Fig. 7C–E). Overexpression of miR-98 attenuated the deterioration of left ventricular performance, as indicated by the increased EF and FS (Fig. 7C–E).

## Discussion

Cardiomyocyte apoptosis has been well documented in viable myocardial areas after MI in experimental and human ischemic heart failure, and suggested as a predominant factor leading to ventricular dysfunction and remodeling^19, 20^. To inhibit myocardial apoptosis and the associated heart diseases, it is crucial to clarify the underlying molecular mechanisms and identify effective therapeutic targets. MiR-98 was introduced into this study due to its close correlation with apoptosis and myocardial dysfunction according to the previous reports^17, 21^. However, the role of miR-98 in MI-induced cardiomyocyte apoptosis remains unknown. Our work demonstrated that miR-98 was upregulated in MI mice and in oxidative stress-stimulated cardiomyocytes. Overexpression of miR-98 attenuated apoptosis in H_2_O_2_-treated NRVCs and MI mice model. Fas and caspase-3 expression were also involved in this research because they were the key modulators of apoptosis and can be regulated by miR-98^17, 18^. In this study, we found that Fas and caspase-3 were negatively regulated by miR-98. In addition, miR-98 targeted at the ACUACCUC sequence in the 3′-UTR of Fas mRNA directly to reduce Fas protein production. Consequently, we acknowledged from this study that miR-98 could negatively regulate MI injury-induced cell apoptosis possibly through Fas and caspase-3 pathway.

There are two major signaling pathways for the regulation of apoptosis. The first pathway is intrinsic pathway, also called ‘mitochondrion pathway’, which has been shown to play a critical role in apoptosis^22^. The other is extrinsic pathway, which concerns membrane-bound death receptors, such as Fas/Fas-L^23^. We investigated whether the two apoptosis pathways were involved in the miR-98-mediated cardioprotection at the same time. Firstly, to investigate the effects of miR-98 on mitochondrial protection, we analyzed the expression of Bcl-2 and Bax and the mitochondrial membrane potential (Δψm). Bcl-2 could prevent the release of cytochrome C from the mitochondria to the cytoplasm, and thus inhibit cell apoptosis^24^. On the contrary, Bax could antagonize the function of Bcl-2 and therefore accelerate cell apoptosis^24^. The intrinsic pathway relies on anti-apoptotic Bcl-2 and pro-apoptotic Bax proteins at mitochondria to sense stress, signal and execute apoptosis of the cell^25, 26^. The current results showed that overexpression of miR-98 reversed the reduction in Bcl-2 expression caused by acute ischemia, suggesting that Bcl-2 is involved in miR-98-induced cardioprotection. Meanwhile, miR-98 reduced the activation of Bax. A reduction in the Δψm is regarded as a hallmark of the early apoptotic period. The results show that the exposure of NRVCs to H_2_O_2_ caused a significant increase of JC-1 monomeric cells relative to that of the control group. By contrast, the number of JC-1 monomeric cells was markedly reduced in NRVCs overexpressed miR-98. Therefore, we have demonstrated for the first time that miR-98 protects against H_2_O_2_-induced mitochondrial dysfunction in NRVCs.

Another mechanism of apoptosis in MI model is via signaling by death receptor members, such as Fas/Fas-L^22, 25, 27^. Fas receptor mediated apoptosis has been reported in experimental myocardial infarction and in chronic heart failure^27, 28^. Fas activation-induced cardiomyocytes apoptosis is also a critical mediator of MI^28^. Enhanced expression of Fas-receptor may result in increasing response to myocardial injury, causing increased apoptosis. The present results showed that the expression of Fas was dramatically upregulated in the infarcted myocardium, the results are in accord with the previous study^28^. At the same time, miR-98 significantly reversed the expression of Fas protein. Sequential activation of caspases plays a central role in the execution-phase of cell apoptosis. In general, the pro-apoptotic members of caspases-8 were looked as the initiators of apoptosis and caspase-3 was the executioners of apoptosis^29^. Caspase-3 has been confirmed as a dominant executor in the Fas death pathway, which results in DNA degradation and apoptosis^30^. Thus inhibition of the activity or function of caspase-3 may depress apoptosis^31^. Western blot showed that the expression caspase-3 was significantly decreased by miR-98. In addition, knockdown of Fas has been shown can significantly reduce Bax expression and increase Bcl-2 expression, which means the correlation between canonical apoptotic pathway and Fas/FasL pathway^32^. Therefore, we speculated that miR-98 simultaneously modulated of the intrinsic and extrinsic pathways of myocardial apoptosis in MI. However, the mechanism by which miR-98 reduced the apoptosis of cardiomyocytes through targeting Fas/Caspase-3 and simultaneously regulating mitochondrial apoptotic pathway remains to be elucidated.

In total, the present study demonstrates that miR-98 suppresses the apoptosis of cardiomyocytes, reduces the MI size, and improves the cardiac function. The cardioprotective effect of miR-98 was achieved by regulating Fas/Caspase-3 apoptotic signal pathway. This reveals that overexpression of miR-98 during the infarct period might be a useful approach for heart protection.

## Methods

### Animals

Healthy adult male Kunming mice (25–30g) used in the present study were kept under standard animal room conditions (temperature, 23 ± 1 °C; humidity, 55 ± 5%) with food and water ad libitum for 1 week before the experiments. The study was approved by the Animal Care and Use Committee of Harbin Medical University. All experimental procedures were performed in accordance with the Guide for the Care and Use of Laboratory Animals, published by the US National Institutes of Health (NIH Publication, 8th Edition, 2011).

### MI Model and Administration of miR-98 agomir

The miR-98 agomirs (Ribo-bio, Guangzhou, China) are double-stranded RNA analogues identical to the mature mmu-miR-98–5p (5′-UGAGGUAGUAAGUUGUAUUGUU-3′). The construct was chemically modified and conjugated with cholesterol moiety for in vivo applications with long-lasting stability and enhanced target specificity and affinity. Before surgery, mice were anesthetized 2, 2, 2-Tribromoethanol (20 mg/kg) and ventilated. The chest was opened via the fourth intercostal space. The ascending aortic artery and the main pulmonary artery were clamped; then, miR-98 agomir (200 nmol·kg^−1^ at the volume of 80 μL) was injected into the left ventricular cavity through the tip of the heart with a 30-gauge syringe. The arteries were occluded for 10 seconds after injection. Mice in sham and MI groups underwent the same procedures but received 80 μL saline. Then MI was induced by ligation of the left anterior-descending (LAD) artery as described previously^19^. In brief, the standard limb lead ECG was continuously recorded on a recorder (BL-420, Taimeng, Chengdu, China). The heart was exposed through a left thoracotomy in the fourth intercostal space and the LAD artery was then ligated with 8–0 sutures was then looped around the LAD coronary artery. Sham-operated mice underwent an identical procedure except that the suture was passed around the vessel without LAD occlusion.

### Measurement of infarct size

Three days after MI, the hearts were harvested and infarct size was measured by TTC (triphenyltetrazolium chloride, Sigma-Aldrich) staining as described previously^17, 33^. After washing out remaining blood and trimming out the right ventricle, the left ventricle was cut into 2-mm thick slices and stained with 1% TTC at 37 °C for 20 minutes, and the infarct area was stainless while the live area turned red. The infarct area were calculated using Image ProPlus 5.0 software (Media Cybernetics, Wokingham, UK). For further study, the tissues in ischemic area of the hearts were collected and stored at −80 °C.

### Echocardiographic measurements

Three days after MI, cardiac function was examined by transthoracic echocardiography with an ultrasound machine (Panoview β1500, Cold Spring Biotech, Taiwan, China) equipped with a 30-MHz phased-array transducer. M-mode tracings were used to measure percentage of ejection fraction (EF%) and fractional shortening (FS%) as described previously^11^.

### Neonatal rat ventricular myocytes culture and transfection

Neonatal rat ventricular cardiomyocyte (NRVCs) from 1 to 3-day-old SD rats were isolated and cultured as described previously^10, 11^. Briefly, the hearts were aseptically removed and ventricle tissues were minced and digested in 0.25% trypsin solution. Dispersed cells were suspended in DMEM (HyClone, Logan, UT) containing 10% fetal bovine serum and centrifuged at 1000 rpm for 5 min and resuspended in medium for 2 h. The isolated cells were plated into culture flasks (noncoated) and 0.1 mmol/l bromodeoxyuridine was added into the medium to deplete nonmyocytes. Cardiomyocytes were cultured at 37 °C with 5% CO_2_ and 95% air. MiR-98-mimic, miR-98 inhibitor and NC were synthesized by Guangzhou RiboBio (Guangzhou, China). Cardiomyocytes were starved in serum-free medium for 24 hours, and then transiently transfected with miR-98 mimic (50 nM), miR-98 inhibitor (100 nM) and NC (50 nM), using X-treme GENE siRNA transfection reagent (Roche, Penzberg Germany) according to the manufacturer’s instructions. Forty-eight hours after transfection, neonatal rat ventricular myocytes were subsequently treated with 100 μM hydrogen peroxide (H_2_O_2_) for 4 h.

### RNA extraction and Real-time PCR

Total RNA was extracted from cultured NRVCs after different treatments or heart tissues using Trizol reagent (Invitrogen, USA) according to manufacturer’s protocols. The levels of miR-98, caspase-3 and Fas mRNA were determined using SYBR Green incorporation on Roche Light-Cycler 480 Real Time PCR system (Roche, Germany), with U6 as an internal control for miR-98 and GAPDH for caspase-3 and Fas. The sequences of primers used were listed as follows: miR-98 F: 5′-GCTGAGGTAGTAAGTTGTATTG-3′; R: 5′-CAGTGCGTGTCGTGGAGT-3′; U6 F: 5′-GCTTCGGCACATATACTAAAAT-3′; R: 5′-CGCTTCACGAATTTGCGTGTCAT-3′; Fas (mouse) F: 5′-TGCTCAGAAGGATTATATCAAGGAG-3′ and R: 5′-CGGGATGTATTTACTCAAGCTAAGA-3′; Fas (rat) F: 5′- TGACTGCTACTGTGGAGAAGAC −3′ and R: 5′- TCATCGCTGAACGCTACTGG -3′; Capase-3 (mouse) F: 5′-CTCGCTCTGGTACGGATGTG-3′ and R: 5′-TCCCATAAATGACCCCTTCATCA-3′; Capase-3 (rat): F: 5′-ATGTCGATGCAGCTAACC-3′ and R: 5′-GTCTCAATACCGCAGTCC-3′. GAPDH (mouse) F: 5′-AAGAAGGTGGTGAAGCAGGC-3′ and R: 5′-TCCACCACCCAGTTGCTGTA-3′; GAPDH (rat): F: 5′-GGAAAGCTGTGGCGTGAT-3′; R: 5′-AAGGTGGAAG AATGGGAGTT-3′. Quantitative real-time PCR was performed in 20 μL volumes with SYBR Green PCR Master Mix (Roche, USA) at 95 °C for 10 min and 40 cycles at 95 °C for 15 s, 60 °C for 30 s and 72 °C for 30 s, using Light Cycler 480 (Roche, USA). The amount of target (2^−ΔΔCT^) was obtained by normalizing to endogenous reference and relative to a calibrator (average of the control samples).

### MTT Assay

Cell viability was assessed using MTT (3-[4, 5-dimethylthiazol-2-yl]-2, 5 diphenyl tetrazolium bromide) assay. Briefly, cells were treated as described above (transfection and H_2_O_2_ treatment, etc.) for indicated time. Next, 20 μL of MTT (0.5 mg/ml) was added into each well for an incubation of 4 h. The supernate was discarded, followed by the addition of 150 μL of DMSO (dimethyl sulfoxide) into each well with rotation for 10 min to dissolve the formazan. The absorbance was measured at 490 nm using an Infinite M200 microplate reader (Tecan, Salzburg, Austria).

### TUNEL staining

The TdT-mediated dUTP nick end labeling (TUNEL) staining was employed to detect the apoptosis in NRVCs and left ventricles (border zones) using a TUNEL fluorescence FITC kit (Roche, USA) according to the manufacturer’s instruction. After TUNEL staining, the cardiomyocytes or the ventricular specimens were immerged into DAPI (1:30, Beyotime Biotechnology, China) solution to stain nuclei. Fluorescence staining was viewed by a Laser Scanning Confocal Microscope (FV1000, Olympus, Japan). The apoptotic rate was calculated as TUNEL-positive cells per field.

### Annexin V-FITC/propidium iodide (AV/PI) dual staining

The Annexin V-FITC/propidium iodide (AV/PI) Apoptosis Detection kit (Vazyme, Nanjing, China) was also used to examine early apoptosis (Annexin V-FITC+/PI−, Q4), late apoptosis (Annexin V-FITC+/PI+ , Q2), and necrosis (Annexin V-FITC−/PI+ , Q1) according to the manufacturer′s instructions (Vazyme, Nanjing, China). To perform the AV/PI staining procedure, as we described previously, cells were digested with 0.25% trypsin, washed, dual-stained with AV and PI, and then analyzed by flow cytometry (BD Bioscience, USA).

### Western Blot Analysis

Total protein was extracted from the NRVCs or peri-infarct region of left ventricular myocardium using RIPA buffer (Sigma-Aldrich, St. Louis, MO, USA) with rotation on ice for 1 h lysis. Subsequently, proteins were separated by electrophoresis on SDS-PAGE (10% polyacrylamide gels) and transferred to nitrocellulose membrane. Next, nitrocellulose membranes were blocked in 5% nonfat milk PBS for 2 hours and then incubated overnight at 4 °C with anti-Fas (1:1000, Abcam, USA), anti-Caspase-3 (1:1000, Cell Signaling Technology, USA), anti-Bcl-2 (1:1000, Cell Signaling Technology, USA), anti-Bax (1:1000, Proteintech, USA) or β-actin (1:1000, ZSGB-Bio, China) primary antibodies, followed by incubation with IRDye secondary antibodies (LI-COR) for 1 hour. The images were captured by the Odyssey CLx Infrared Imaging System (LI-COR Biosciences, Lincoln, NE, USA). Western blot bands were quantified by measuring the intensity in each group using Odyssey CLx version 2.1. The data was normalized to β-actin as an internal control.

### Luciferase reporter assay

The rat Fas 3′-UTR (GenBank ID: NM_139194.2, nt 961–1141) was cloned into the multiple cloning site of the pmirGLO dualluciferase miRNA target expression vector (Promega, Madison, WI, USA), referred to as pmiRGLO-Fas. Then, HEK293T cells were seeded in a 96-well plate and co-transfected with 0.5 μg plasmid and miR-98 mimics or negative controls using Lipofectamine 2000 reagent. Renilla luciferase was used as an internal control. Forty-eight hours after transfection, the cells were collected, and firefly and Renilla luciferase activities were evaluated using Dual-Luciferase Reporter Assay System (Promega, Madison, WI, USA).

### Caspase-3 and LDH activity assay

Myocardial caspase-3 activity and serum LDH activity were determined by colorimetric assay kits (Beyotime Institute of Biotechnology, Jiangsu, China; Nanjing Jiancheng Bioengineering Institute, Nanjing, China) as described in our previous study^11^. The heart tissue and blood samples were collected from mice 3 days after MI and the activity of Caspase-3 and LDH were measured with the colorimetric method according to the manufacture’s protocols, respectively.

### Statistical analysis

All data were presented as mean ± SEM and analyzed by SigmaPlot and SigmaStat Software (Jandel Scientific, CA. USA). Paired t test or Student’s t test was used where appropriate. A two-tailed P < 0.05 was considered to be statistically significant.

## Electronic supplementary material

Supplementary Information
