# Supplementary material for: MicroRNA-98 negatively regulates myocardial infarction-induced apoptosis by down-regulating Fas and caspase-3
Source: Sci Rep. 2017 Aug 7;7:7460. doi: 10.1038/s41598-017-07578-x (PMC5547114; doi:10.1038/s41598-017-07578-x)
Supplement: Supplementary file 1 — Supplementary Information [file 41598_2017_7578_MOESM1_ESM.pdf]

MicroRNA-98 negatively regulates myocardial infarction-induced apoptosis by down-regulating Fas and caspase-3

Chuan Sun[1#], Huibin Liu[1#], Jing Guo[1], Yang Yu[1], Di Yang[1], Fang He[1], Zhimin Du[1]*

[1]Institute of Clinical Pharmacology of the Second Affiliated Hospital (Key Laboratory of Drug Research, Heilongjiang Higher Education Institutions), Harbin Medical University, Harbin 150086, Heilongjiang Province, P. R. China

[#]These authors contributed equally to this work.

*Correspondence: Zhi-Min Du, Institute of Clinical Pharmacology of the Second Affiliated Hospital, Harbin Medical University, Xuefu Road 246, Nangang District, Harbin 150086, Heilongjiang Province, P. R. China

Tel/Fax: +86-451-86605353

Email: dzm1956@126.com

Supplementary Information

Supplementary Figures

Supplementary Figure S1

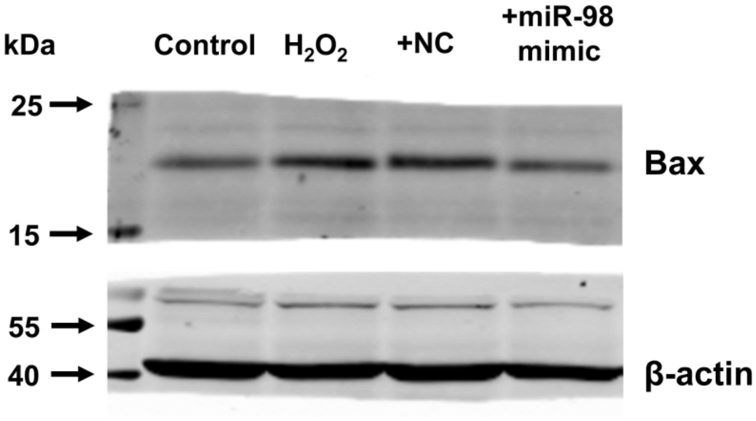

Legend S1. Full-length blots of Figure 3(A) in the main text. Effect of miR-98 on Bax expression. NC indicates miRNA negative control, +NC indicates co-application of H_2O_2 and NC, +miR-98 mimic indicates co-application of H_2O_2 and miR-98 mimic.

Supplementary Figure S2

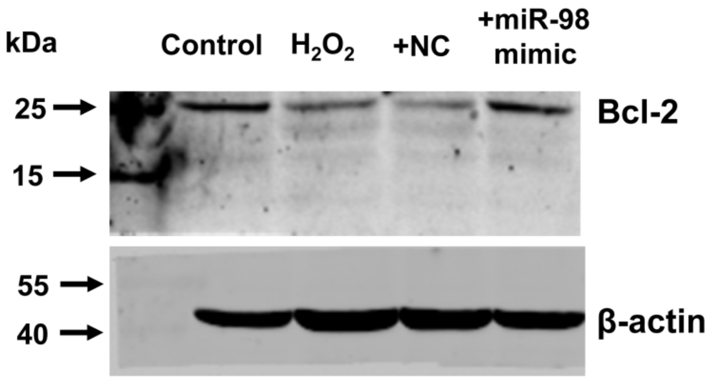

Legend S2. Full-length blots of Figure 3(B) in the main text. Effect of miR-98 on Bcl-2 expression. NC indicates miRNA negative control, +NC indicates co-application of H_2O_2 and NC, +miR-98 mimic indicates co-application of H_2O_2 and miR-98 mimic.

Supplementary Figure S3

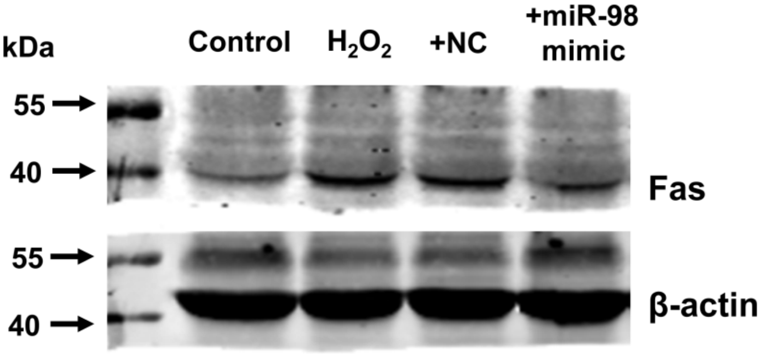

Legend S3. Full-length blots of Figure 4(B) in the main text. Effect of miR-98 on Fas expression. NC indicates miRNA negative control, +NC indicates co-application of H_2O_2 and NC, +miR-98 mimic indicates co-application of H_2O_2 and miR-98 mimic.

Supplementary Figure S4

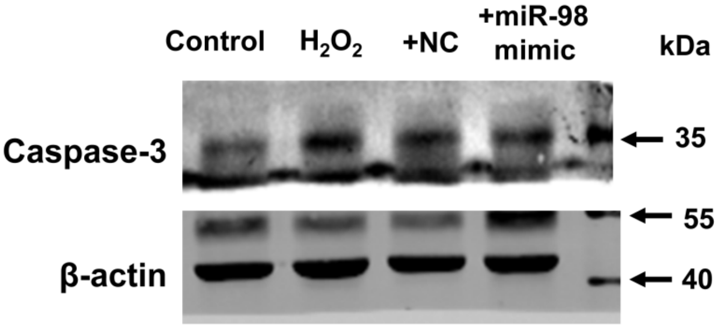

Legend S4. Full-length blots of Figure 4(D) in the main text. Effect of miR-98 on Caspase-3 expression. NC indicates miRNA negative control, +NC indicates co-application of H_2O_2 and NC, +miR-98 mimic indicates co-application of H_2O_2 and miR-98 mimic.
